# Assessment of leukemia inhibitory factor and glycoprotein 130 expression in endometrium and uterine flushing: a possible diagnostic tool for impaired fertility

**DOI:** 10.1186/1472-6874-12-10

**Published:** 2012-04-20

**Authors:** Manal A Tawfeek, Manal A Eid, Azza M Hasan, Manal Mostafa, Hesham A El-Serogy

**Affiliations:** 1 Faculty of Medicine, Department of Clinical Pathology, Tanta University, Tanta 31111, Egypt; 2 Faculty of Medicine, Department of Microbiology and Immunology, Tanta University, Tanta 31111, Egypt; 3 Faculty of Medicine, Department of Gynecology and Obstetrics, Tanta University, Tanta 31111, Egypt

**Keywords:** Leukemia inhibitory factor, Glycoprotein 130, Impaired fertility

## Abstract

**Background:**

Uterine receptivity and implantation are complex processes requiring coordinated expression of molecules by zygote and uterus. Our objective was to evaluate the role of the endometrial expression of leukemia inhibitory factor (LIF) and its glycoprotein 130 (gp130) receptor molecules and their secretion in uterine flushing during the window of implantation in cases of primary unexplained infertility

**Case presentation:**

The study was conducted on 25 infertile women with unexplained infertility for at least two years and 10 normal fertile women as a control group . Endometrial tissue and uterine flushing were obtained. Each tissue specimen was divided into two pieces; one piece was used for histological dating of the endometrium and for immunostaining of progesterone receptors, and the second was used for RNA extraction and PCR assay of LIF and gp130 mRNA expression. Serum estrogen and progesterone were measured for all subjects. LIF mRNA was expressed in the endometrium of all normal fertile women but significantly decreased in infertile women. LIF was not detectable in 88% of infertile women while it was fairly detectable in 12% of them. Gp130 mRNA was hardly detectable in both fertile and infertile women with no difference between them. Infertile women secreted significantly less LIF and gp130 molecules in the uterine flushing compared with normal fertile women.

**Conclusions:**

Expression of LIF mRNA in endometrium could be used as a molecular marker of unexplained infertility. Assessment of secreted LIF and gp130 molecules in uterine flushing could be another useful and safe method for predicting successful implantation as well as for diagnosing and eventually treating women with impaired fertility using recombinant human LIF.

## Background

One in ten couples has problems conceiving. Of these, 25% have unexplained infertility [1]. Embryo implantation is a critical step in establishment of pregnancy. Implantation is the process by which the blastocyst becomes intimately connected with the maternal endometrium (decidua). In humans, the process of implantation can be divided into three phases: apposition, adhesion, and invasion. The opposition comprises the blastocyst orientation in the uterine cavity towards the endometrium. During the adhesive and invasive phases, the blastocyst approaches the epithelium, attaches itself to it, and the embryo trophoblast invades the deciduas. All these steps are controlled by a variety of interacting molecules of both maternal and embryonic origin [2]. Many cytokines, growth factors and adhesive molecules are known to trigger the initial process of implantation during the adhesive phase, and realize embryo–maternal contacts during the invasive phase [1].

The endometrium becomes receptive for a limited period of time after exposure to 17-β-estradiol (E) followed by progesterone. Embryo transfer studies have identified a phase of uterine receptivity, the “window of implantation,” between days 5 and 10 following the luteinizing hormone (LH) surge [3]. Many factors produced by the endometrium during this window have been proposed as molecular markers of a receptive endometrium. While the progesterone receptor (PR) is the only factor as yet identified to be absolutely required for successful implantation, leukemia inhibitory factor (LIF) and mucin 1 are regarded as two of the most important signaling vectors [4]. Leukemia inhibitory factor is a pleiotropic cytokine of the IL-6 family; i.e., it has effects on many different cell types and that its activities are not restricted to one lineage [5]. It is a polyfunctional, highly glycosylated protein (Mw 38–67 KDa) that mediates a wide range of effects in different ways, from promotion of cell proliferation and lifespan, to the control of their differentiation with regard to the tissue environment in which the target cells perform their functions. Mature LIF protein of maternal origin and its encoding mRNA have been identified in endometrium. Human fallopian tubes also express LIF; its mRNA is detectable throughout the cycle [6] and is expressed in cervical mucus [7]. In the endometrium, LIF expression remains low during the proliferative phase, rises after ovulation and remains high until the end of each menstrual cycle with its maximal expression during the mid-late secretory phase. Its high endometrial production in the middle and late phases of menstrual cycle stresses its important role in implantation [5].

Simultaneously, the blastocyst expresses LIF receptor (LIF-R), indicating the significance of LIF in embryo–maternal crosstalk. Endometrium and oocyst also express LIF-R [8]. In uterine flushing samples, LIF is measured to predict reproductive outcomes [9]. Other cells that express LIF-R include neurons, megakaryocytes, macrophages, adipocytes, hepatocytes, osteoblasts, myeloblasts, and kidney and breast epithelial cells. The LIF-R complex includes two subunits: the LIF-specific subunit LIFR-β, and the glycoprotein 130 (gp130) subunit (also used by IL-6 and IL-11) [8].

The gp130 subunit is a transmembrane protein. Soluble forms of gp130 are generated by proteolytic cleavage or by alternative splicing. Binding of LIF to its receptor promotes formation of a receptor complex with gp130. Signal transduction involves activation of members of the Janus family and phosphorylation of members of the STAT family of transcription factors. The LIFR-β/gp130 heterodimer can bind and signal in response to oncostatin M, ciliary neurotrophic factor, and cardiotrophin, in addition to LIF. Binding of LIF to LIF -R and gp130 activates signal transduction pathways [10].

The biological activity of LIF and IL-6 is affected by levels of their soluble (s) receptors: sIL-6R, sLIFR, and sgp130. The IL-6/sIL6R complex is able to bind and signal through membrane-bound gp130. Soluble gp130 acts as an antagonist—preventing the cytokine/receptor complex from initiating signals through membrane-bound gp130. In consequence, the ratio of LIF/sgp130 and IL-6/sgp130 is altered in endometrium of infertile women. Infertility in such cases appears to be associated with a failure by endometrium to express the normal molecular repertoire characteristic of the receptive period. Reduced sgp130 secretion may be a new molecular marker of such dysregulation in women with unexplained infertility [10]. In humans, LIF can be detected in samples from both endometrial biopsies and uterine flushing. The majority of women with unexplained infertility had dysregulated LIF production. Likewise, endometrial explants derived from infertile women showed reduced levels of LIF secretion [11]. Based on these finding, this study was designed to investigate the expression of LIF and its receptor subunit gp130 in endometrium of infertile women.

## Case presentation

### Patient selection

The study was conducted on 25 infertile women with unexplained infertility for at least 2 years, and 10 normal fertile women as a control group. The inclusion criteria were 2 years of primary unexplained infertility which was diagnosed by the following; presence of regular menstrual cycle between 24 and 35 days, absence of tubal or ovarian pathology proved by laparoscopy and hysterosalpingo-contrast sonography, absence of endometriosis proved by laparoscopy, absence of ovulatory disorders proved by folliculometry and normal serum progesterone level, normal semen analysis according to WHO criteria, normal thyroid function and normal plasma prolactin concentration. The mean age of the infertile women was 31.1 ± 4.2 years (range; 24–39 years). The mean age of the fertile women was 30.7 ± 6.5 years (range; 20–38 years). Each of the fertile women had at least one live birth, without history of infertility or miscarriage. None of the participants had taken steroids or other medications for at least 2 months prior to collection of samples. The study was carried out under the guidance of the Ethics and Research Committee of Tanta Faculty of Medicine.

### Materials

Samples of endometrial tissue and uterine flushing were obtained from enrolled subjects after obtaining their informed consent. Endometrial tissue was collected by obtaining small strips of endometrium using Novak’s curette.

Uterine flushing was performed as described previously [12]. The uterine cervix was visualized with a speculum, the anterior cervical lip was grasped and the cervical os was cleaned with sterile dry gauze. A bimanual pelvic examination was not done before introduction of the flushing device. A 14-gauge Foley 3-way balloon catheter (Cat. no. 41-825-10: Eschmann, Shoreham-by-Sea, UK) was wrapped in a polythene sheath (width 19 mm, thickness 30 mm; Mackinnon & Hay, Edinburgh), which was heat-sealed at one end, to avoid contamination by endocervical mucus. The rigidity required for introduction of the device was effected by an intraluminal metal introducer (diameter 1 mm; length 42 cm, excluding the circular distal grip made to prevent perforation of the catheter by the introducer). When the tip of the device had reached the uterine fundus, the sheath was withdrawn, thus breaking the heat-seal. The metal introducer was removed and the balloon was inflated within the uterine cavity with 1 ml sterile 0.154 M sodium chloride solution. This amount was selected because it is well below the volume of the uterus and was sufficient to retain the catheter and avoid leakage during flushing. Because there could be no loss of fluid through the occluded Fallopian tubes, the quantity of fluid recovered always exceeded 95% of the initial flushing volume

Immediately after collections the flushing sample was transferred into a tube containing EDTA. This aliquot was twice frozen and thawed to effect hemolysis. The remaining fluid was centrifuged at 1800 g for 10 min at 4°C. The supernatant was stored at −20°C until analysis [13].

The tissue and flushing samples were stored at −20°C for LIF and gp130 assays. All specimens were collected during the implantation window (LH surge: LH + 6 to LH + 11) [10]. Each tissue specimen was divided into two pieces. One piece was used for obtaining tissue sections for histological dating of the menstrual cycle by a pathologist according to the method of Noyes et al. [14], and for PR immunostaining. The second piece was used for RNA extraction, and PCR assays of LIF and gp130 mRNA expression. Uterine flushing samples were used for estimation of LIF and gp130 secretion by enzyme-linked immunosorbent assay (ELISA). Venous blood samples were collected from all women participating in the study for measurement of serum estrogen and progesterone. Expression of *LIF* and *gp130* mRNA in endometrium was assayed using RT-PCR technique.

### RNA extraction and cDNA synthesis

Total cellular RNA was extracted from endometrial tissue using Qiagen RNeasy mini-spin column (RNeasy Mini Kit, Qiagen, USA) according to the manufacturer protocol. Complementary DNA was prepared from the RNA as follows: 2 μg of total RNA was reverse-transcribed with random hexamers by use of a commercial kit (High-Capacity cDNA kit, Applied Biosystems, USA) under the following conditions: hexanucleotides annealing for 10 min at 25°C, cDNA synthesis for 30 min at 48°C, followed by enzyme inactivation for 5 min at 95°C.

### cDNA amplification

The cDNA was used as a template to amplify LIF and the two splice variants of LIF -R subunit gp130. The amplification mixture was performed in a final volume of 50 μl containing 5 μl cDNA, 25 μl Taq PCR master mix (2.5 units Taq DNA polymerase, 1× PCR buffer, and 200 μM of each dNTP) (Taq PCR Master Kit, Applied Biosystems, USA), and 200 μM of each primer (Table 1). The PCR entailed an initial denaturation at 95°C for 5 min; followed by 35 cycles of: 1-minute denaturation at 94°C, 1-minute annealing at 63°C, and 1-minute extension at 72°C; followed by a final extension at 72°C for 5 min (for LIF), 30 min denaturation at 94°C, 30-minutes annealing at 57°C, and 1-minute extension at 72°C, followed by a final extension at 72°C for 5 min (for gp130 A and B). Amplification of gp130 using primers C and D was performed in the same way except that the primer annealing temperature was 50°C [10,15]. As an internal control, GAPDH was amplified to identify differences in RNA input and reverse transcription efficiency. Amplification of GAPDH was performed for each sample in a separate tube using the following primers and probe: forward primer: 5′-GAAGGTGAAGGTCGGAGTC-3′, reverse primer: 5′-GAAGATGGTGATGGGATTTC-3′. The PCR conditions for GAPDH were an initial denaturation at 95°C for 5 min’; followed by 35 cycles of 45-second denaturation at 94°C, 45-second annealing at 62°C, and 1-minute extension at 72°C, followed by a final extension at 72°C for 5 min. The amplified DNA was fractionated on 1% agarose gel and photographed. The densities of the LIF and gp130 bands were divided by the density of GAPDH of the same sample to get normalized expression values. 

**Table 1:** Primer sequences used in the study (10 & 15)

**Primer**	**Location**	**Sequence (5′ to 3′)**
LIF sense	3174-3195	CAGCATCACTGAATCACAGAGC
LIF antisense	3712-3733	CCCTGTGGGGATGTTTCATACT
Splice variant 1		
gp130 A	927-946	ATACTGGAGTGACTGGAGTG
gp130 B	1099-1118	CATCTTGTGAGAGTCACTTC
Splice variant 2		
gp130 C	1767-1790	GGTACGAATGGCAGCATACA
gp130 D	2480-2461	CTGGACTGGATTCATGCTGA

### Measurement of LIF and gp130 in uterine flushing

Levels of LIF and gp130 were measured in uterine flushing samples using the LIF ELISA kit, Bender Medsystems, USA and the Quantikine ELISA kit, R&D Systems Inc., respectively. The sensitivity of the LIF assay ranges from 0.45–500 pg/ml; that for gp130 ranges from 40–6000 pg/ml. The procedures were performed according to the manufacturers’ instructions; fresh saline was used as zero standards in both estimates.

### Expression of LIF and gp130 mRNA in endometrium

**Figure 1:**
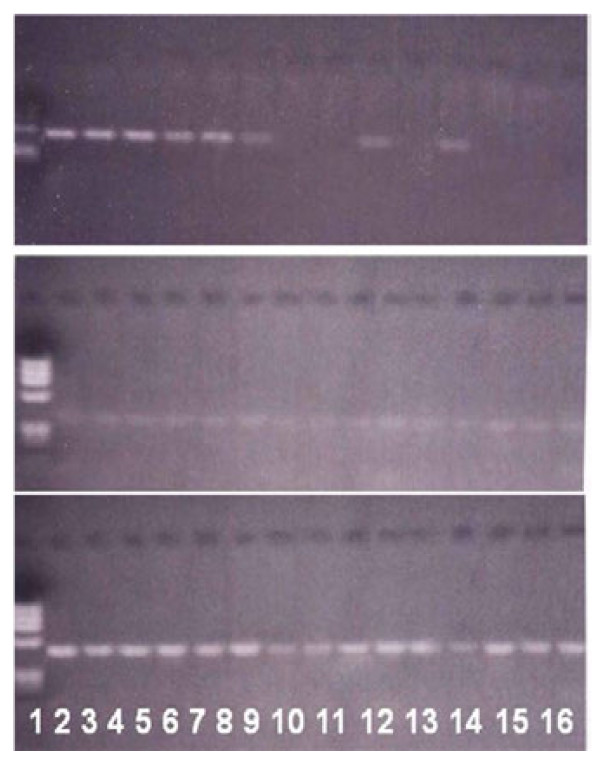
**Expression of*LIF*, and*gp130*splice variant 2 in endometrium of fertile and infertile women.** The upper figure represents *LIF* expression, middle figure represents expression of gp130 and the lower one is the *GAPDH* expression. Lane 1 is the DNA ladder (100 bp); lanes from 2–5 show samples from fertile women and lanes 6–16 show samples from infertile women.

Leukemia inhibitory factor and mRNA of the two gp130 splice variants were measured in samples of endometrial tissue taken from fertile and infertile women during the implantation window (LH surge LH + 6–LH + 13) using RT-PCR. The fertile and infertile groups enrolled in the study were matched as regards subjects’ ages, plasma hormonal profiles (FSH, LH, estrogen, progesterone and prolactin), and endometrial PR expression (Table 2; Figure 1). Expression of LIF in endometrium was significantly lower in infertile women compared with fertile women (*P* < 0.001). All fertile women showed marked *LIF* mRNA expression in endometrium. Only 3 (12%) of the 25 infertile women enrolled in the study showed detectable endometrial LIF expression; 22 patients (88%) did not showed any *LIF* mRNA in their endometrial samples.

**Table 2:** Comparison of plasma hormones, endometrial progesterone receptors and LIF and gp130 expression in endometrium and uterine flushing samples from fertile and infertile women

	**Fertile**	**Infertile**	**Significance,*P***
Age (mean ± SD)	30.7 ± 6.5	31.1 ± 4.2	>0.05
Prolactin (mean ± SD)	13.1 ± 5.6	11.7 ± 4.4	>0.05
FSH in Serum	5.6 ± 1.8	6.9 ± 1.4	>0.05
LH in serum	9.3 ± 4.0	8.1 ± 5.1	>0.05
Estradiol in serum	9.1 ± 3.3	10.5 ± 4.0	>0.05
Progesterone receptor in endometrium	Expressed in 100%	Expressed in 100%	>0.05
LIF			
­In uterine flushing	48.8 ± 28.9	3.9 ± 7.5	<0.001*
­In endometrium	Expressed in 100%	Expressed in 12%	<0.001*
gp130			
­In uterine flushing	182 ± 77	51.5 ± 27.5	<0.001*
­In endometrium	Expressed in 70%	Expressed in 76%	>0.05

* denotes statistical significance.

An RT-PCR analysis of the two *gp130* splice variants showed very faint expression in both fertile and infertile women, with no difference between the two groups (*P* > 0.05). In the fertile group, 3 (30%) women did not show any gp130 splice variant expression; 7 (70%) women showed very low expression of splice variant1. Similarly, 6 (24%) of the 25 infertile women showed complete absence of *gp130* mRNA expression and 19 (76%) showed very faint expression of gp130 variant 1 (Figure 1).

### Secretion of LIF and gp130 in uterine flushing

Uterine flushing was performed using 2 ml saline. The procedure was done very gently to avoid pain. Absence of pain is an essential feature of this exploration since pain may enhance cytokine production and thereby produce inconsistent results. Estimation of LIF and gp130 concentrations in flushing samples was performed using ELISA assays (Table 2).

Secretion of LIF in uterine flushing samples was significantly lower in infertile women than in fertile women (*P* < 0.001). In fertile women, uterine flushing LIF levels ranged from 18–120 pg/ml (mean: 48.8.2 ± 28.9). In infertile women, uterine flushing LIF levels varied between 0.5 and 35 pg/ml (mean: 3.9 ± 7.5). Similarly, secretion of gp130 in uterine flushing was significantly lower in the infertile group (mean: 51.5 ± 27.5, range; 25–140 pg/ml) than in the fertile group (mean: 182 ± 77, range; 95–370 pg/ml) (*P* < 0.001; Table 2).

## Conclusions

The implantation process is considered the most relevant limiting factor for successful pregnancy. Molecules that affect uterine receptivity for subsequent implantation of the blastocyst include integrins, colony stimulating factor-1 (CSF-1) and LIF. The fact that LIF is the key molecule in the implantation process in monkeys and mice inspired similar research in humans, which demonstrates that *LIF* mRNA is present in endometrium, and that increased LIF expression coincides with the implantation window. LIF belongs to the IL-6 family of cytokines, which signal through specific receptors on the cell surface that all share gp130 subunit as a common accessory signal transduction molecule [16].

The role of endometrial LIF in implantation has been documented in animals and human. LIF has been also found in the fallopian tube during preimplantation period, indicating a role for LIF in communication between the embryo and the tube. This observation suggests that absence of LIF in the mother, rather than the embryo, is responsible for failure of implantation [17,18]. We started our study by confirming the expression of *LIF* mRNA in the endometrium of both fertile and infertile women during the implantation window. Expression of *LIF* mRNA in the endometrium was significantly reduced in infertile women compared with the fertile group. Dimitriadis et al. reported that immunostaining of endometrial tissue from fertile and infertile women for IL-11, IL-11Rα, and LIF —molecules affecting implantation—demonstrated equal staining for IL-11 and its receptor, but marked reduction of LIF staining in infertile women compared with the fertile group [19]. Mutations in the *LIF* gene have been described in nulligravid infertile women, and have been hypothesized to cause transcription abnormalities and decreased LIF expression [16,20].

Measurement of LIF secretion in uterine flushing samples as a noninvasive technique enables the determination that low LIF concentration in such fluid during late luteal phase could be predictive of implantation failure [19]. The present study shows that secretion of LIF in samples of uterine flushing of infertile women during window of implantation was markedly low. Several studies have reported lower LIF in uterine flushing samples from women with primary unexplained infertility than in fertile women, and lower LIF secretion from endometrial explants of infertile women than fertile ones, especially during the implantation window [11,19,21]. However, these results contrast with those of Ledee-Bataille et al., who showed an inverse correlation between LIF concentrations in uterine flushing samples and the likelihood of successful implantation [22]. The conflict between the different reports might be due to the use of different ELISA kits. Reportedly, commercially available ELISA kits are far from equivalent, and mainly differ in their ability to detect glycosylated protein. Human LIF is known to be highly glycosylated; the sugar moiety represents as much as 50% of the total weight of the naturally produced protein. Several peptide epitopes may therefore be masked and the protein may not be detectable by the antibodies in certain kits [16].

While presence of sufficient amount of LIF protein seems to be an essential condition for implantation, a variety of other regulatory mechanisms also affect implantation, such as malfunction of LIF receptor, IL-6 signal transducer (gp130), which is an affinity modulator for the LIF protein receptor complex [16]. In a previous study, sgp130 was the most abundant member of the IL-6 cytokine-receptor family secreted by the endometrium at the time of implantation [10]. Therefore, the present study focuses on exploration of gp130 status in both endometrium and uterine flushing. The study showed minimal expression of gp130 splice variant1 in the endometrium of both fertile and infertile women equally. However, infertile women secreted significantly reduced levels of sgp130 in uterine flushing samples taken during the implantation period. Sherwin et al. demonstrated very weak gp130 immunostaining of endometrial tissue during the secretary phase of menstrual cycle [10]. Moreover, cultured endometrial biopsies taken from infertile women secreted significantly less gp130 than fertile biopsies did. Depending on that, the biological activity of LIF in the endometrium is affected by the levels of their soluble gp130 receptor. They reported that the reduced secretion of gp130 by infertile women during the widow of implantation points to a functional difference in endometrium at that time. Counter-intuitively, fertile women secrete higher soluble gp130 in uterine flushing samples in the presence of gp130 mRNA expression as low as that found in infertile women; this is apparently due to soluble gp130 being produced by proteolytic cleavage of the membrane-bound receptor.

In conclusion, the study indicates the possibility that endometrium of women with idiopathic infertility has abnormalities in expression and secretion of important cytokines as LIF and its gp130 receptor molecules, which may contribute to altered uterine receptivity and so infertility. The study also suggests that uterine flushing may help to detect the unreceptive uterus before assisted reproduction treatments and in the future, help to verify the normalization of cytokine concentrations to improve uterine receptivity before ovarian stimulation.

## Abbreviations

CSF-1, Colony stimulating factor-1; E, 17-β-estradiol; ELISA, Enzyme-linked immunosorbent assay; gp130, glycoprotein130; LH, Luteinizing hormone; LIF, Leukemia inhibitory factor; LIF-R, LIF receptor; mRNA, Messenger RNA; NaCl, Sodium chloride; PR, Progesterone receptor; S, Soluble.

## Competing interests

The authors declare that they have no competing interests.

## Authors’ contributions

The following authors were responsible for study concept and design: MAT, MAE, HE, MM and AMH take responsibility for acquisition of data. All authors were responsible for analysis, interpretation of data, drafting of the manuscript, critical revision of the manuscript and statistical analysis. All authors confirm that they have read and approved the final manuscript.

## Pre-publication history

The pre-publication history for this paper can be accessed here:

http://www.biomedcentral.com/1472-6874/12/10/prepub
